# Analysis of Differences in Metabolite and Antioxidant Activity in Highland Red Raspberry Pulp Based on Widely Targeted Metabolomics

**DOI:** 10.3390/molecules30102124

**Published:** 2025-05-11

**Authors:** Yangbo Song, Jie Wang, Xiaojian Pu

**Affiliations:** 1 College of Agriculture and Animal Husbandry, Qinghai University, Xining 810016, China; 2021990010@qhu.edu.cn; 2 Academy of Animal Science and Veterinary, Qinghai University, Xining 810016, China; wangjie08142023@163.com; 3 Key Laboratory of Northwest Cultivated Land Conservation and Marginal Land Improvement Enterprises, Ministry of Agriculture and Rural Affairs, Delingha 817000, China

**Keywords:** plateau raspberry, fruit pulp, widely targeted metabolomics, characteristic metabolites, antioxidant capacity

## Abstract

In order to achieve differentiated utilization of red raspberry fruit pulp, a widely targeted metabolomics analysis of pulp from two regions was performed to explore the effect of plateau environment on the accumulation of secondary metabolites of red raspberry. Ultra-high performance liquid chromatography–mass spectrometry (UHPLC-MS/MS), combined with principal component analysis and Orthogonal Partial Least Squares Discriminant Analysis (OPLS-DA), was used to process the data and correlate them with the results of four antioxidant assays. Fourteen metabolites were characterized in the fruit pulp of Qinghai raspberries, and 618 up-regulated differential metabolites were found, which was 4.35 times higher than that of Yunnan. Flavonoids and phenolic acids were more abundant, with kaempferol-3-O-sambubioside being endemic to Qinghai, and saccharin-7-*O*-glucoside and rhamnocereus citrinus being endemic to Yunnan. Metabolic pathway enrichment analysis showed that the fruit pulp from the two regions differed significantly (*p* < 0.01) in ATP-binding cassette transporter (ABC transporter), purine metabolism, and so on. Antioxidant analysis showed that the Yunnan raspberries (Y-RP) were significantly superior to Qinghai raspberries (Q-RP) in terms of DPPH radical scavenging ability (DPPH) and ferric ion reducing/antioxidant power (FRAP), while Q-RP was significantly superior to Y-RP in terms of oxygen radical absorbance capacity (ORAC) and ABTS radical scavenging capacity (ABTS). This study showed that the plateau environment significantly promotes the accumulation of functional secondary metabolites of red raspberry, which provides a theoretical basis for the development of the functional components of plateau raspberry.

## 1. Introduction

Raspberry (*Rubus idaeus* L.), commonly referred to as red raspberry or mountain berry, is a deciduous perennial shrub that produces fruit. Its fruits exhibit a variety of colors, including red, black, and purple-red, and are noted for their appealing appearance as well as their richness in natural functional components such as polyphenols, flavonoids, polysaccharides, and terpenes [1,2]. The provinces of Yunnan and Qinghai are situated in high-altitude regions, both exceeding 2000 m above sea level. Despite the harsh climatic conditions, their unique climate features (long hours of sunlight, strong radiation, significant diurnal temperature variations, and mineral-rich soil) facilitate the diverse formation and accumulation of secondary metabolites in raspberries [3]. In traditional Chinese medicine, raspberries are believed to possess several beneficial properties, including antibacterial, anti-inflammatory, anti-tumor, antioxidant, anti-allergic, liver-protective, and analgesic effects [4,5,6,7]. Studies have indicated that raspberries may improve gut health and reduce the risk of heart disease [8,9]. Current research has primarily emphasized the nutritional components of raspberries and the comparison and exploration of their functional components [10,11]. However, there is comparatively less research on the accumulation of secondary metabolites in raspberries from high-altitude regions and the differences in characteristic metabolites.

The differences in plant metabolites are primarily influenced by genetic and environmental factors [12,13]. Marić et al. [14] employed ultra-high-performance liquid chromatography-triple quadrupole-time of flight mass spectrometry (UHPLC-Triple-TOF-MS) technology to examine the seed metabolites of various raspberry varieties, elucidating their differences in polyphenolic metabolic mechanisms. Wang [15] and Zhang [16] investigated the impact of environmental factors on Ningxia goji berries and the effects of drought stress on licorice metabolites with ultra performance liquid chromatography tandem mass spectrometry (UPLC-MS/MS) technology. Research on raspberries primarily focuses on the physiological characteristics of the fruit trees, their basic nutritional components, and common bioactive ingredients, particularly the identification and purification methods of active substances such as organic acids, along with their current applications in the pharmaceutical and food industries [17]. Other studies have also examined the differences in nutritional quality of red raspberry seeds [18], the variations in metabolic components between mature fruits of red and yellow raspberries [19], and the patterns of phenolic content and antioxidant enzyme activity during the growth of red raspberry leaves [20]. However, research on the metabolite composition and antioxidant properties of raspberry pulp in high-altitude regions remains insufficient.

This study employed UPLC-MS/MS-based, widely targeted metabolomics technology and antioxidant testing to conduct a comparative analysis of the metabolites in red raspberry pulp sourced from high-altitude regions in Yunnan and Qinghai. The aim was to explore the impact of the plateau environment on the metabolites, metabolic pathways, and antioxidant properties of raspberry pulp. Additionally, the study seeks to identify key secondary metabolites that significantly contribute to the antioxidant properties in both regions, reveal the metabolic differences in plateau red raspberries from different origins, and provide theoretical references for the development and utilization of plateau raspberries.

## 2. Results

### 2.1. Metabolite Annotation and Comparative Analysis of Metabolite Profiles

A total of 1336 secondary metabolites across 12 categories were detected in Yunnan raspberry pulp (Y-RP) and Qinghai raspberry pulp (Q-RP) using ultra-high performance liquid chromatography-tandem mass spectrometry (Table S1). Among these, Y-RP contained 1322 secondary metabolites, while Q-RP contained 1333. The two shared 1319 functional components, accounting for 99.77% of Y-RP and 98.95% of Q-RP, respectively. Only three secondary metabolites were found to be unique to Yunnan raspberry pulp, while 14 were found to be unique to Qinghai raspberry pulp (Table 1). The types of metabolites in the raspberry pulps from Yunnan and Qinghai were highly similar; however, the Qinghai raspberry pulp exhibited a slight advantage in the relative abundance of metabolites.

**Figure 1:**
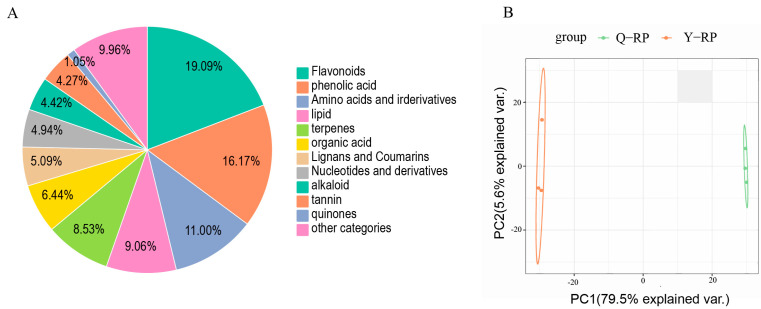
Metabolomics profiles of Y-RP and Q-RP. (**A**) Classification of the annotated metabolites in Y-RP and Q-RP. (**B**) Principal component analysis (PCA) results show metabolite profile differences between and within groups.

The detected metabolites were classified into 12 major categories. Among these, flavonoids were the most abundant, comprising 255 metabolites (19.09%), followed by phenolic acids with 216 metabolites (16.17%). Amino acids and their derivatives accounted for 147 metabolites (11.00%), lipids for 121 metabolites (9.06%), and terpenoids for 114 metabolites (8.53%) (Figure 1A). The PCA results indicated significant differences in the metabolite profiles between Y-RP and Q-RP (Figure 1B). The first principal component explained 79.5% of the total variance, while the second principal component accounted for 5.6%, demonstrating that these components effectively distinguished between the different samples.

### 2.2. OPLS-DA, and Permutation Test of Raspberry Pulp from Two Regions

**Figure 2:**
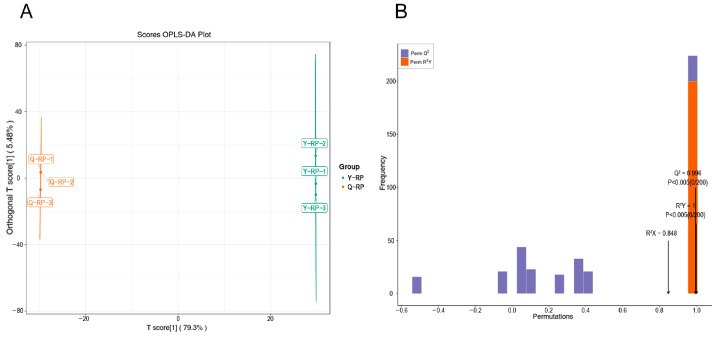
Analysis of OPLS-DA. (**A**) OPLS-DA score plots. (**B**) OPLS-DA Validation plots.

Orthogonal Partial Least Squares Discriminant Analysis (OPLS-DA) is a regression modeling method that analyzes multiple dependent variables against multiple independent variables [21]. This method is highly effective in differentiating pairwise comparisons and enhancing the validity and resolution of the model. In this study, OPLS-DA was employed to analyze the 1336 metabolites, with the contribution rates of the first and second principal components in the OPLS-DA score plot being 79.3 and 5.48%, respectively (Figure 2A). The OPLS-DA model was utilized to compare the metabolite composition of Y-RP and Q-RP, yielding values of R^2^X = 0.848, R^2^Y = 1, and Q^2^ = 0.996 (Figure 2B). The high values of R^2^X, R^2^Y, and Q^2^ indicated that these analyses are reproducible, reliable, and suitable for screening differential metabolites.

### 2.3. Screening of Differential Metabolites

**Figure 3:**
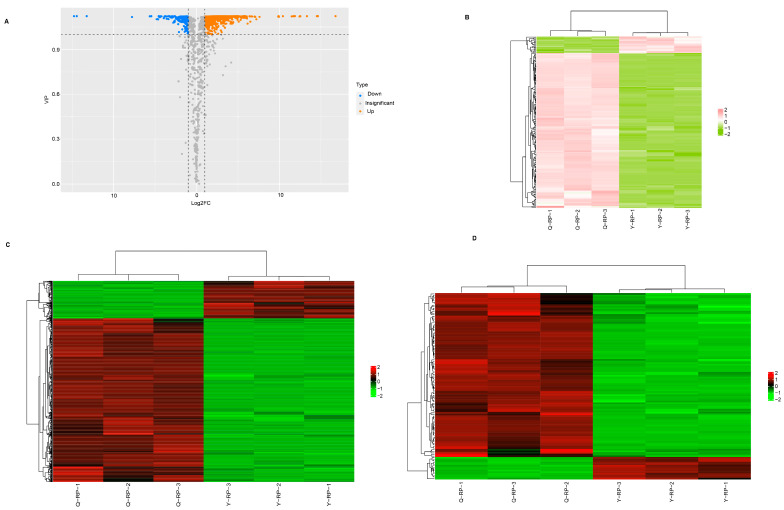
Analysis of differential metabolites in fruit pulps between Yunnan and Qinghai. (**A**) Volcano plot of differential metabolites. (**B**) Clustering heatmap of differential flavonoid metabolites. (**C**) Clustering heatmap of differential metabolites. (**D**) Clustering heatmap of the contents of differential phenolic acid metabolites.

Based on the results of Orthogonal Partial Least Squares Discriminant Analysis (OPLS-DA), univariate statistics, including Fold Change (FC) and Variable Importance in Projection (VIP), were employed for metabolite quality control and the identification of differential metabolites (screening criteria: FC ≥ 2 or ≤ 0.5, VIP ≥ 1). The differential levels of metabolites in raspberries are illustrated in Figure 3A,C. Table 2 enumerates the 760 differential metabolites identified, which account for 56.89% of the total metabolite types.

This includes 166 flavonoids (21.84%), 116 phenolic acids (15.26%), 75 terpenoids (9.87%), 69 lipids (9.08%), 66 amino acids and their derivatives (8.68%), 48 nucleotides and their derivatives (6.32%), 42 lignans and coumarins (5.53%), 39 tannins (5.13%), 37 organic acids (4.87%), 26 alkaloids (3.42%), 8 quinones (1.05%), and 68 other substances (8.95%). Among the 760 differential metabolites, 618 (81.32%) were found to be upregulated in Q-RP. Flavonoids and phenolic acids represented the two categories of metabolites with the most significant differences.

### 2.4. Major Differential Metabolites in Raspberry Pulp from Yunnan and Qinghai

A total of 255 flavonoids were detected in the two types of pulp, among which 166 were identified as differential metabolites. This group includes 10 chalcones, 22 flavanones, 11 flavanonols, 10 anthocyanins, 32 flavones, 64 flavonols, and 17 flavanes. Of these 166 compounds, 150 were upregulated in Q-RP, while 16 were upregulated in Y-RP. The characteristic flavonoid compounds in Q-RP include kaempferol-3-*O*-sambubioside (log2FC = 16.76) and kaempferol-3-*O*-(6’‘-malonyl) glucoside-7-*O*-glucoside (log_2_FC = 12.52). By contrast, the characteristic flavonoid functional components in Y-RP are eriodictyol-7-*O*-glucoside (log_2_FC = −14.77) and rhamnocitrin (log_2_FC=−13.24) (Figure 3B). Additionally, phenolic acids constitute the second major class of differential metabolites in the fruit pulp of raspberries from Yunnan and Qinghai (Table 2). The clustering heatmap of differential phenolic acid metabolites clearly illustrates the differences between the two regions (Figure 3D). A total of 216 phenolic acid compounds were detected, of which 116 exhibited significant differences; notably, 102 phenolic acid compounds were significantly upregulated in Q-RP (Table 2).

**Figure 4:**
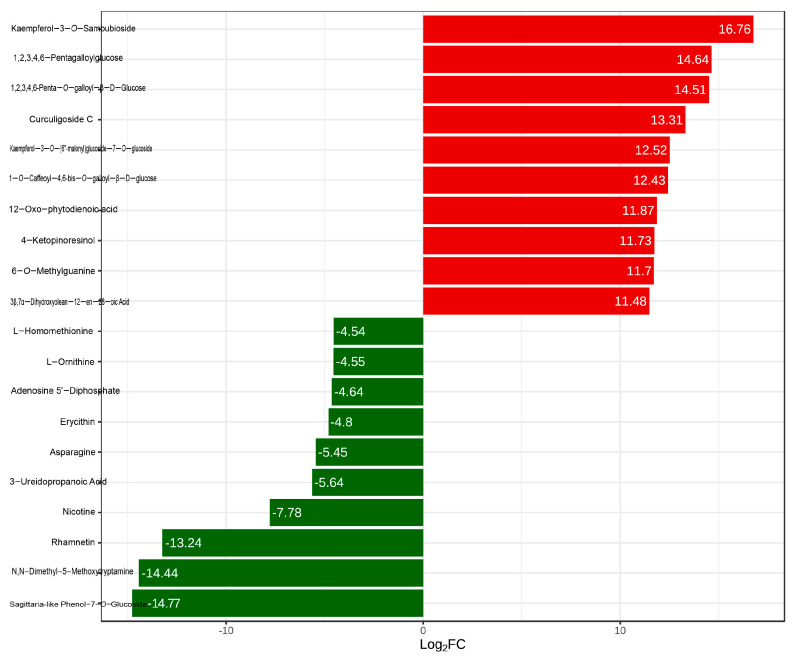
Main differentially expressed metabolites (Top 10).

The top ten differential metabolites were identified based on the Fold Change (Figure 4). In Q-RP, the metabolites exhibiting a significantly higher relative content compared to Y-RP include two phenolic acids (1,2,3,4,6-penta-*O*-galloyl-β-D-glucose and 1-*O*-caffeoyl-4,6-di-*O*-galloyl-β-D-glucose), one nucleotide and its derivative (6-*O*-methylguanine), two flavonoids (kaempferol-3-*O*-sambubioside and kaempferol-3-*O*-(6’‘-malonyl)glucoside-7-*O*-glucoside), one lignin and coumarin (4-ketopinoresinol), two tannins (1,2,3,4,6-pentagalloylglucose and curculigoside C), one terpenoid (3β,7α-dihydroxyolean-12-en-28-oic acid), and one lipid (12-oxo-phytodienoic acid). Conversely, in Q-RP, the metabolites with a significantly lower relative content compared to Y-RP consist of three amino acids and their derivatives (L-asparagine, L-ornithine, and L-homomethionine), one nucleotide and its derivative (adenosine-5’-diphosphate), three flavonoids (eriodictyol-7-*O*-glucoside, rhamnocitrin, and rhodionin), two alkaloids (N,N-dimethyl-5-methoxytryptamine and nicotine), and one organic acid (3-ureidopropionic acid) (Figure 4). All the differential metabolites are detailed in Table S2.

### 2.5. Main Metabolic Pathways Involved in Differential Metabolites of Raspberry Pulp in Yunnan and Qinghai

**Figure 5:**
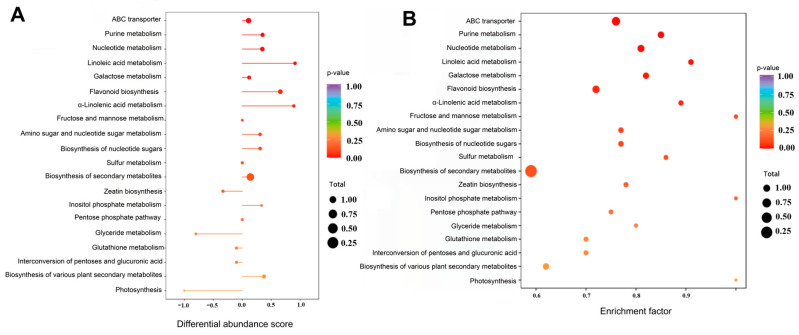
KEGG enrichment analysis. (**A**) Differential abundance score plot. (**B**) Enrichment bubble plot.

By comparing the 760 differential metabolites of Q-RP and Y-RP with the KEGG database, 88 metabolic pathways closely related to the different expressions were identified through enrichment analysis. Seven significantly different metabolic pathways (*p* < 0.05) were selected and ranked from the smallest to the largest P value: ABC transporters, purine metabolism, nucleotide metabolism, linoleic acid metabolism, galactose metabolism, flavonoid biosynthesis, and α-linolenic acid metabolism (Figure 5). KEGG pathway annotation analysis indicated that the pathways related to purine metabolism and flavonoid biosynthesis are the primary differential metabolic pathways associated with antioxidant activity. Notably, the biosynthesis of secondary metabolites and the biosynthesis of various plant secondary metabolites significantly impact antioxidant activity.

### 2.6. Correlation of Antioxidant Capacity Between Y-RP and Q-RP

**Figure 6:**
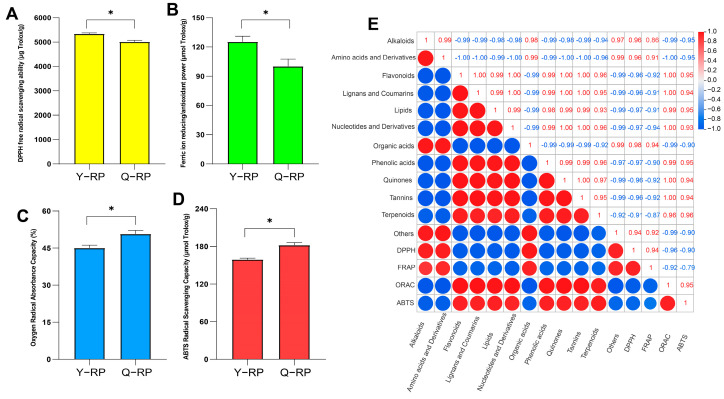
Analysis of the antioxidant capacity of red raspberry fruit pulps from the two regions and correlation analysis of metabolites. (**A**) Assay of DPPH free radical scavenging ability (DPPH). (**B**) Assay of ferric ion reducing/antioxidant power (FRAP). (**C**) Assay of oxygen radical absorbance capacity (ORAC). (**D**) Assay of ABTS radical scavenging capacity (ABTS). (**E**) Correlation analysis of metabolites. * represents a significant difference (*p* < 0.05).

The DPPH free radical scavenging ability (Figure 6A) and ferric ion reducing/antioxidant power (FRAP) (Figure 6B) of the Y-RP group were both higher than those of the Q-RP group, and there were significant differences (*p* < 0.05). Conversely, the oxygen radical absorbance capacity (ORAC) (Figure 6C) and ABTS radical scavenging capacity (ABTS) (Figure 6D) of the Q-RP group were both higher than those of the Y-RP group, and there were significant differences (*p* < 0.05). There was a correlation between the antioxidant activities of raspberry fruit pulps (0.1 g) from the two regions and the differential metabolites (Figure 6E). DPPH showed a positive correlation with alkaloids, amino acids and their derivatives, organic acids, and other categories, while there was a significant negative correlation between DPPH and compounds such as lipids, nucleic acids, and their derivatives. FRAP showed a positive correlation (*p* > 0.95) with alkaloids, amino acids and their derivatives, organic acids, and other categories, and a negative correlation with the remaining compounds. ABTS and ORAC showed a positive correlation with flavonoids, lignins and coumarins, lipids, nucleotides and their derivatives, phenolic acids, quinones, tannins and terpenoids, and a negative correlation with the remaining compounds.

## 3. Discussion

In the evolutionary process of adapting to the plateau environment, plants have gradually enhanced their metabolism and the production of various metabolites [22,23]. Due to the unique growing conditions of the plateau, raspberries have facilitated the accumulation of specific secondary metabolites, including flavonoids, phenolic acids, terpenoids, and tannins. This study indicates that Qinghai raspberry pulp is particularly enriched with unique metabolites, especially flavonoids. The number of unique metabolites identified in Qinghai raspberry pulp is significantly higher, with 14 distinct compounds compared to only 3 in Yunnan raspberry pulp. In Yunnan raspberry pulp, the flavonoids include eriodictyol-7-*O*-glucoside and rhamnocitrin. Among these, eriodictyol-7-*O*-glucoside is known for its potent free radical scavenging capabilities [24], while rhamnocitrin exhibits notable pharmacological activity [25]. By comparison, the unique metabolic components in Qinghai raspberry pulp consist of 14 types, including 2 flavonoids, 2 phenolic acids, 2 tannins, 4 lipids, and 1 each of terpenes, lignans, coumarins, nucleotides and their derivatives, and alkaloids. Among these, kaempferol-3-*O*-sambubioside is a flavonoid involved in the formation of the blue color in plant petals [26], which has shown antioxidant, anti-inflammatory, and antidiabetic potential [27,28], and 1,2,3,4,6-penta-*O*-galloyl-β-D-glucose is a significant compound for radiation resistance and free radical scavenging [29].

In Qinghai raspberry pulp, lipid components that regulate plant stress responses—such as 12-oxo-phytodienoic acid—and 6-*O*-methylguanine, which is involved in DNA damage repair, have been identified [30,31,32]. The tannins, specifically curculigoside C and 1,2,3,4,6-penta-*O*-galloylglucose, exhibit significant antioxidant and neuroprotective effects [33,34]. Although these components have not been extensively documented in existing studies, their presence may hold considerable significance for the antioxidant properties of raspberries and their adaptability to high-altitude environments. Previous research has indicated that Codonopsis from different regions displays notable differences in the types and levels of metabolites, resulting in variations in quality [35]. This study examined the influence of production regions on metabolite differences by identifying metabolites in raspberry pulps from the Qinghai and Yunnan plateaus. The results revealed that flavonoids and phenolic acids exhibited distinct clustering patterns across different regions [36], particularly in Q-RP, which demonstrated a richness of characteristic functional components among the top 10 differential metabolites.

Further KEGG pathway enrichment analysis revealed significant enrichment in the synthesis of bases and glycosides, flavonoid biosynthesis, and the metabolism of linoleic acid and α-linolenic acid in Q-RP, which is closely related to its unique growth environment. Studies indicate that different cultivation regions and climatic conditions significantly affect the metabolic components of raspberries. Drought and low-temperature environments induce plants to produce large amounts of reactive oxygen species, while flavonoids and phenolic acids act as antioxidants, effectively scavenging these reactive oxygen species and protecting plant cells from oxidative damage [37].

By comparing the differential metabolites of flavonoids and phenolic acids in raspberry pulp from Qinghai and Yunnan, it was found that the flavonoid content in Qinghai raspberry pulp is higher, with the variety reaching 150 types, while the phenolic acid variety also amounts to 102 types. This discovery indicates that Q-RP significantly outperforms Y-RP in ORAC and ABTS antioxidant capacities. Further analysis revealed a significant positive correlation between phenolic acids and ORAC (*p* < 0.05) [38]. Additionally, organic acids have a substantial impact on the scavenging effect of DPPH radicals [39]. Salicylic acid, which is abundant in raspberries, can induce resistance in fruits and vegetables against pathogens, increasing the levels of peroxidase and catalase. These antioxidant enzymes directly or indirectly utilize phenolic substances as substrates [40]. In this experiment, salicylic acid (log_2_FC = 1.77) and methyl salicylate-2-*O*-glucoside (log_2_FC = 1.05) were found in Qinghai raspberry pulp. Although the DPPH capacity of Q-RP was lower than that of Y-RP, organic acids showed a significant positive correlation with DPPH (*p* < 0.05). It was indicated that non-phenolic antioxidants play a crucial role in enhancing the overall antioxidant activity of plants. Extracts of non-phenolic components such as terpenoids and polysaccharides, in plants still exhibit strong antioxidant activity even when the content of phenolic substances is low [41,42]. Different antioxidants in plants can cooperate with each other through means such as electron transfer and hydrogen atom transfer. For example, in the classic antioxidant combination of vitamin C and vitamin E, vitamin C can re-reduce the oxidized vitamin E, restoring its antioxidant activity. The synergistic effect of the two greatly enhances the antioxidant effect [43]. We speculate that there may also be a similar synergistic pathway between flavonoids and non-phenolic antioxidants in the Y-RP system. Although the level of flavonoids is low, through the synergy with non-phenolic antioxidants, a high DPPH/FRAP activity is still achieved.

In summary, the diverse phenolic compounds present in Qinghai raspberry pulp contribute to its superior ORAC and ABTS radical scavenging capabilities. In contrast, Yunnan raspberry pulp exhibits stronger ferric reducing antioxidant power and DPPH radical scavenging effects. These findings suggest that the variations in the composition and content of secondary metabolites in plateau raspberry pulps from different regions result in differences in their antioxidant properties, with each type exhibiting unique advantages in antioxidant performance.

## 4. Materials and Methods

### 4.1. Materials and Reagents

This experiment utilized high-quality fruits (autumn fruits, identical maturity, proper shape, uniformly and vividly colored peel, free from pests and diseases, and without decay) from commercial cultivars of double-cropping raspberries (‘Heritage’ and *Rubus idaeus* var. *acuminatus*) sourced from Yunnan and Qinghai provinces. The Yunnan raspberries (Y-RP) grow in Zhanyi District, Qujing City, Yunnan Province (103°80′ E, 27°49′ N), at an altitude of 2000 m. The annual sunshine duration is 2098 h. It has a subtropical plateau monsoon climate, with an average annual temperature of 17.4 (16.3–18.6) °C, an average annual precipitation of 1002 m, a frost-free period of 255 d, and the soil type is red soil. The Qinghai raspberries (Q-RP) are produced in Sujiabao, Datong County, Qinghai Province (101°63′ E, 36°80′ N), at an altitude of 2280 m. The annual sunshine duration is 2553 h. It has a plateau continental climate, with an average annual temperature of 4.9 °C, an average annual precipitation of 523 m, a frost-free period of 133 d, and the soil types are alpine rocky soil and alpine meadow soil. Formic acid, acetonitrile, and methanol were all of chromatographic grade, and dimethyl sulfoxide (DMSO) was purchased from Sigma-Aldrich LLC (St. Louis, MO, USA).

### 4.2. Research Methodology

#### 4.2.1. Sample Preparation and Extraction

Raspberries were pulped and filtered to obtain the fruit pulp. The freeze-dried samples were ground in a mixer mill with zirconium oxide beads at a frequency of 30 Hz for 90 s. A total of 50 mg of the freeze-dried powder was dissolved in 1.2 mL of a 70% CH_3_OH solution, with rotation occurring for 30 s every 30 min, culminating in a total of six rotations. After centrifugation at 12,000 rpm for 3 min, the extract was filtered and subsequently used for UPLC-MS/MS analysis. Three biological replicates were used for each group.

#### 4.2.2. LC-ESI-MS/MS Analysis

The raspberry pulp samples were analyzed for their components using a Liquid Chromatography Electrospray Ionization Tandem Mass Spectrometry (LC-ESI-MS/MS) system (LC: ExionLC™ AD; MS: Applied Biosystems 4500 Q TRAP) (Agilent, Santa Clara, California, USA). The analysis conditions were as follows: The LC column used was an Agilent SB-C18 (2.1 mm × 100 mm, 1.8 µm) (Agilent, California, USA.). The mobile phase consisted of solvent A (ultrapure water containing 0.1% formic acid) and solvent B (acetonitrile containing 0.1% formic acid). The sample measurement commenced with 95% A and 5% B as the starting conditions. At 9 min, a linear gradient was applied, changing to 5% A and 95% B, which was maintained for 1 min. Subsequently, the gradient was adjusted back to 95% A and 5.0% B within 70 sec and maintained for an additional 2.9 min. The flow rate was set at 0.35 mL/min, the column oven temperature was maintained at 40 °C, and the injection volume was 4 μL. The effluent was alternately connected to an ESI-QTRAP-MS/MS.

#### 4.2.3. ESI-QTRAP-MS/MS

The operating parameters for the Electrospray Ionization (ESI) source were as follows: the source temperature was set at 550 °C, and the ion spray voltage was 5500 V and −4500 V in positive and negative ion modes, respectively. The ion source gases, Gas I, Gas II, and Curtain Gas, were maintained at pressures of 50, 60, and 25 psi, respectively, while the Collision Activated Dissociation (CAD) was set to high. Triple quadrupole mass spectrometer scanning was used for the multiple reaction monitoring (MRM) experiment. The collision gas (nitrogen) was set at a medium level. The collision energy cycled through 20 -40-60 V. Through further optimization of the declustering potential (DP) and collision energy (CE), the DP and CE values for individual MRM transitions were obtained. The DP ranged from 50 to 120 V, and the CE ranged from 15 to 35 eV. Based on the metabolites eluted during each period, a specific set of MRM transitions was monitored in each period.

#### 4.2.4. Determination of Antioxidant Activity

To determine antioxidant activity, 15 mL of the extract was combined with 15 mL of a 0.1 mmol/L DPPH solution in a test tube. This mixture was incubated in the dark for 30 min. Subsequently, 200 μL of the final mixture was transferred onto a microplate, and the absorbance was measured at 517 nm. For samples exhibiting high antioxidant activity, appropriate dilutions should be performed prior to measurement. An amount of 80% methanol was used as the control, and Trolox was used as the standard. The results are expressed as Trolox equivalents per gram of fresh weight (TE μg/g FW) [44].

The ferric ion reducing antioxidant power (FRAP) was determined by diluting 40 µL of the extract with distilled water at a ratio of 1:20 (*V/V*) and mixing it with 200 µL of freshly prepared tripyridyltriazine reagent (Fe3^+^-TPTZ) in a 96-well microplate. The Fe^3+^-TPTZ reagent was prepared by combining 20 mmol/L of FeCl_3_·6H_2_O, 10 mmol/L of TPTZ, and 40 mmol/L of HCl with 300 mmol/L of acetate buffer in a volumetric ratio of 1:1:10 (*V/V/V*). After incubation in the dark at 25 °C for 30 min, the absorbance was measured at 593 nm, with the average standard deviation (SD) of three replicates expressed as µmol/L Trolox equivalent (TE µmol/L/g FW) per gram of fresh weight [45].

The ABTS radical scavenging activity was assessed using a 96-well microplate by combining 7 mmol/L of ABTS (5 mmol/L of NaH_2_PO_4_, 5 mmol/L of Na_2_HPO_4_, and 154 of mmol/L NaCl) at pH 7.4 with 2.5 mmol/L of potassium persulfate. This mixture was stored in the dark at room temperature for 16 h, after which it was diluted with ethanol to achieve an absorbance of 0.70 ± 0.02 at 734 nm. Subsequently, 15 µL of the extract was mixed with 285 µL of the freshly prepared ABTS solution and incubated in the dark at room temperature for 10 min. A standard calibration curve of Trolox was established at concentrations of 0, 80, 160, 240, 320, and 400 μmol/L. The absorbance values were measured at 734 nm, and the results are expressed in μmol/L Trolox equivalents per gram of Fresh weight (TE μmol/L/g FW) [46].

The oxygen radical absorbance capacity (ORAC) method is based on the hydrogen atom transfer process, during which the fluorescent probe is oxidized by peroxyl radicals. Following the instructions provided by the Oxygen Radical Antioxidant Capacity Assay Kit from CELL BIOLABS, INC (San Diego, CA, USA), ORAC testing was conducted on the fruit pulp. The kit comprises a 96-well microplate with a clean black bottom, a 100× fluorescein probe, a radical initiator, an antioxidant standard (Trolox), and an assay diluent (4×). The sample was prepared by dissolving it at a ratio of 1:100 [47].

### 4.3. Data Analysis

Differences between samples were assessed using one-way analysis of variance (ANOVA), and the False Discovery Rate (FDR) was controlled by the multiple testing correction method of Benjamini-Hochberg (BH). In this study, the FDR threshold was set at 0.05. Metabolites with a corrected *p*-value (*p* < 0.05) were considered as significantly differential metabolites. Data analysis and graphical generation were performed using SIMCA 14.2, R Studio 4.2.3, and the Metware cloud platform (Maiwei Metabolism, Wuhan, China) (https://cloud.metware.cn/) (accessed on 23 May 2022). Metabolomics data were processed using multivariate statistical analysis methods, including Principal Components Analysis (PCA), Orthogonal Partial Least Squares-Discriminant Analysis (OPLS-DA), and Hierarchical Cluster Analysis (HCA). The identification and quantification of metabolites were based on the MWDB and the corresponding methods established by Maiwei Biotechnology Co., Ltd. Additionally, pathway information for the metabolites was obtained from the Kyoto Encyclopedia of Genes and Genomes (KEGG) database. The annotated results were subsequently subjected to enrichment analysis to identify differential metabolic pathways. Finally, the relevant data of the oxidation capacity determination were analyzed using GraphPad Prism 8.0.1 software.

## 5. Conclusions

Using UPLC-MS/MS-based widely targeted metabolomics technology, we detected 1336 metabolites in the fruit pulp of highland red raspberries from Yunnan and Qinghai, of which 760 were identified as differential metabolites, accounting for 56.89% of the total metabolite species. Significant differences were observed in the metabolic composition of the fruit pulp between Yunnan and Qinghai, with Q-RP exhibiting a richer variety of metabolites; the number of upregulated metabolites in Q-RP was 4.35 times greater than that in Y-RP. Three unique metabolites were identified in the fruit pulp of Yunnan red raspberries, while 14 unique metabolites were found in Qinghai red raspberries. Metabolic pathway analysis revealed pathways related to purine metabolism and flavonoid biosynthesis. Multiple functional components, such as flavonoids and phenolic acids, were enriched, and the differences between these two regions contribute to the varying antioxidant capacities observed across different origins. Therefore, by fully leveraging the differences in antioxidant suitability of raspberry pulp between Yunnan and Qinghai, functional products with varying emphases can be developed.

## Figures and Tables

**Table 1:** Statistics of metabolites in raspberry fruit pulp.

Type	Total	Common	Y-RP	Q-RP
Flavonoids	255	251	Holy Grassinol-7-*O*-Glucoside, Rhamnus Limonin	Holy Grassinol-7-*O*-Glucoside, Rhamnus Limonin
Phenolic acid	216	214	-	1,2,3,4,6-penta-*O*-galloyl-β-D-glucose, 1-*O*-caffeoyl-4,6-bis-*O*-galloyl-β-D-glucose
Amino acids and Irderivatives	147	147	-	-
Lipid	121	117	-	2-linoleyl glycerides-1,3-di-*O*-glucoside, 1-linoleyl glycerides-2,3-di-*O*-glucoside, 2-alpha-linolenic acid glycerides-1-*O*-glucoside, 12-oxo-Phytodienoic acid
Terpenes	114	113	-	3β, 7α-Dihydroxyoleanum-12-ene-28-acid
Organic acid	86	86	-	-
Lignans and Coumarins	68	67	-	4-Ketoeugenol
Nucleotides and Derivatives	66	65	-	6-*O*-Methylguanine
Alkaloid	59	57	N, N-Dimethyl-5-Methoxytryptamine	Isobutyryl carnitine
Tannin	57	55	-	Citronin C, 1,2,3,4,6-pentagalaryl glucose
Quinones	14	14	-	-
Other Categories	133	133	-	-
Total	1336	1319	3	14

Notes: “-” means not detected. Y-RP and Q-RP represent the fruit pulp of raspberries from Yunnan and Qinghai, respectively.

**Table 2:** Statistical table of different metabolite species in Yunnan and Qinghai raspberry pulp.

Type	Total Number of Detections	Total Number of Differential Metabolites	Up-Regulated Metabolites in Y-RP	Up-Regulated Metabolites in Q-RP
Flavonoids	255	166	16	150
Phenolic acid	216	116	14	102
Terpenes	114	75	4	71
Lipid	121	69	4	65
Amino acids and Irderivatives	147	66	32	34
Nucleotides and Derivatives	66	48	13	35
Lignans and Coumarins	68	42	7	35
Tannin	57	39	2	37
Organic acid	86	37	12	25
Alkaloid	59	26	14	12
Quinones	14	8	1	7
Other categories	133	68	23	45
Total	1336	760	142	618

## Data Availability

The original contributions presented in this study are included in the article/Supplementary material. Further inquiries can be directed to the corresponding author.

## Supplementary Materials

The following supporting information can be downloaded at: https://www.mdpi.com/article/10.3390/molecules30102124/s1, Table S1: The types and contents of compound; Table S2: All the differential metabolites.
